# Whole Genome Transcript Profiling of Drug Induced Steatosis in Rats Reveals a Gene Signature Predictive of Outcome

**DOI:** 10.1371/journal.pone.0114085

**Published:** 2014-12-03

**Authors:** Nishika Sahini, Saravanakumar Selvaraj, Jürgen Borlak

**Affiliations:** Centre for Pharmacology and Toxicology, Hannover Medical School, Hannover, Germany; University of Basque Country, Spain

## Abstract

Drug induced steatosis (DIS) is characterised by excess triglyceride accumulation in the form of lipid droplets (LD) in liver cells. To explore mechanisms underlying DIS we interrogated the publically available microarray data from the Japanese Toxicogenomics Project (TGP) to study comprehensively whole genome gene expression changes in the liver of treated rats. For this purpose a total of 17 and 12 drugs which are diverse in molecular structure and mode of action were considered based on their ability to cause either steatosis or phospholipidosis, respectively, while 7 drugs served as negative controls. In our efforts we focused on 200 genes which are considered to be mechanistically relevant in the process of lipid droplet biogenesis in hepatocytes as recently published (Sahini and Borlak, 2014). Based on mechanistic considerations we identified 19 genes which displayed dose dependent responses while 10 genes showed time dependency. Importantly, the present study defined 9 genes (ANGPTL4, FABP7, FADS1, FGF21, GOT1, LDLR, GK, STAT3, and PKLR) as signature genes to predict DIS. Moreover, cross tabulation revealed 9 genes to be regulated ≥10 times amongst the various conditions and included genes linked to glucose metabolism, lipid transport and lipogenesis as well as signalling events. Additionally, a comparison between drugs causing phospholipidosis and/or steatosis revealed 26 genes to be regulated in common including 4 signature genes to predict DIS (PKLR, GK, FABP7 and FADS1). Furthermore, a comparison between *in vivo* single dose (3, 6, 9 and 24 h) and findings from rat hepatocyte studies (2 h, 8 h, 24 h) identified 10 genes which are regulated in common and contained 2 DIS signature genes (FABP7, FGF21). Altogether, our studies provide comprehensive information on mechanistically linked gene expression changes of a range of drugs causing steatosis and phospholipidosis and encourage the screening of DIS signature genes at the preclinical stage.

## Introduction

Hepatic steatosis or non-alcoholic fatty liver disease (NAFLD) evolves from excessive intracellular lipid accumulation in the form of cytosolic lipid droplets. It can be induced by a broad spectrum of conditions including overnutrition, diabetes and drug treatment [1]. The frequency of drug induced steatosis is unknown and estimates of drug induced hepatic injury range considerably amongst different studies and countries with a crude incidence rate of 19.1/100,000 as was recently reported for the general population of Iceland [2]. Owing to its anatomical location and biochemical functions the liver plays a key role in the detoxification of drugs and other foreign compounds. Upon prolonged exposure to xenobiotics and as a result of cellular stress and mitochondrial dysfunction its metabolic functions become compromised that can be an entry into micro- and macrovesicular steatosis. Recently, drug induced inhibition of mitochondrial fatty acid oxidation and steatosis has been reviewed [3] and the main mechanism by which drugs cause steatosis can be summarised as

1. Direct inhibition of mitochondrial beta oxidation enzymes
2. Sequestration of CoA and/or L-Carnitine
3. Inhibition of the mitochondrial respiratory chain/mitochondrial membrane potential
4. Impairment of mitochondrial DNA replication
5. Impaired peroxisome proliferator activated receptor (PPARα) transcriptional activity
6. Alterations of other pathways in lipid homeostasis

Importantly, unresolved drug induced steatosis may progress to non-alcoholic steatohepatitis (NASH), fibrosis and further architectural changes of the liver [4]. Microvesicular steatosis, as induced by mitochondrial dysfunction also affects oxidative phosphorylation and beta oxidation of fatty acids to lower cellular ATP pools [3], [5]. Furthermore, excessive reactive oxygen production (ROS) can be provoked by drug metabolism and in conjugation with mitochondrial dysfunction propagates ROS that leads to lipotoxicity, impaired glucose and lipid metabolism and the formation of ectopic LDs [3], [6], [7]. Besides, drugs have the ability to induce phospholipidosis by interfering with lysosomal enzymes [8]–[10] and several *in vitro* studies on drugs/chemicals in causing steatosis and/or phospholipidosis were recently published [11]–[15]. This include studies with amiodarone and tetracycline that induced up-regulation of lipogenic genes in HepaRG cells [13], while in the study of Park and colleagues 12 drugs/chemicals were investigated in HepG2 cells that either caused phospholipidosis and/or steatosis [15] to evidence human hepatoma cells to be useful in preclinical drug testing. So far more than 50 novel chemicals were identified to induce phospholipidosis, however, the true relationship between xenobiotic induced- steatosis and phospholipidosis remains elusive [16]. As drug induced steatosis (DIS) and phospholipidosis (PLD) are common observations in preclinical safety testing and in order to improve an understanding of drug induced steatosis whole genome gene expression profiles in livers of rats treated with a wide range of different drugs were investigated.

Specifically, from our recent publication we identified about 200 genes [17], [18] involved in various pathophysiological events during fatty liver development. In the present study we applied our conceptional thinking on lipid droplet formation to drug induced steatosis and for this purpose analysed whole genome transcript expression data deposited in the publically available Open TG-GATEs database (http://toxico.nibio.go.jp). We studied dosage and time dependant effects of 17 drugs that caused lipid droplet formation as evidenced by histopathology. The animal studies were conducted by the Japanese National Institute of Health Science, National Institute Biomedical Innovation (NIBIO) and 15 pharmaceutical companies in a concerted effort [19] using the following reference chemicals and drugs: carbon tetrachloride (CCL4), hydroxyzine (HYZ), imipramine (IMI), amitriptyline (AMT), ethinylesterdiol (EE) all of which are well known to cause steatosis [20]. Other compounds implicated in fatty liver included methapyrilene hydrochloride (MP), coumarin (CMA), tetracycline (TC), lomustine (LS), vitamin A (VA), diltiazem (DIL), disulfiram (DSF), colchicine (COL), ethionalamide (ETH), ethanol (ETN) and puromycin aminonucleoside (PAN) to provoke steatosis.

The gene expression data were grouped into low, medium and high dose treated animals after single and repeated treatment for up to 28 days. In all data from about 1000 microarray experiments could be retrieved to identify differences and commonalities amongst drugs that may play a role in pathogenesis of fatty liver. A hallmark of LD formation is the expression of perilipin family of proteins which are well known for their ability to associate with LDs in various tissues. Specifically, perilipin 2 and 3 proteins associate with LDs in hepatic steatogenesis [21]. Based on reported works, we predict significant up-regulation of genes coding for these proteins in DIS. Genes commonly regulated after drug administration at all-time points were considered and further categorised based on their biological functions such as lipid transport, lipid synthesis, LD growth, signalling events and glucose metabolism. Our aim was to understand the pathways adapted by different drugs in steatotic conditions for which we present differentially expressed gene (DEG) data as enriched gene networks and gene frequency graphs to analyse the probable roles of these genes in causing drug induced steatosis and to develop a gene signature predictive of drug induced fatty liver outcome.

Furthermore, we considered drugs inducing phospholipidosis that is caused by impaired lysosomal functions leading to accumulation of excess phospholipids in the form of lamellar bodies [8]. Differentiating this condition from the steatosis phenotype by haematoxylin and eosin staining (H and E) proves to be impossible [22]. Hence, molecular markers and genes responsible for each of these conditions as well as genes commonly regulated in both conditions are highly desirable. To resolve this, we compared DEGs from steatotic rat livers with DEGs from drug induced phospholipidosis. Lastly, DEGs identified in single dose treated animals (up to one day) were compared with *in vitro* rat hepatocyte studies and we explored the possibility to predict a gene signature that is common amongst diverse drugs and conditions that can be used for drug screening purposes as to identify culprit drug candidates with risk of causing hepatic steatosis.

## Materials and Methods

### Chemicals

Drugs/and chemicals used for the study include carbon tetrachloride (CCL4), hydroxyzine (HYZ), imipramine (IMI), amitriptyline (AMT), ethinylesterdiol (EE), methapyrilene hydrochloride (MP), coumarin (CMA), tetracycline (TC), lomustine (LS), vitamin A (VA), diltiazem (DIL), disulfiram (DSF), colchicine (COL), ethionalamide (ETH), ethanol (ETN) and puromycin aminonucleoside (PAN) whose dosage, vehicle, route of administration and time points are summarised in Table S1. Note, this data is derived from the TGP-GATE database.

Additionally, chemicals such as haloperidol (HPL); perhexilline (PH); tamoxifen (TMX); ketoconazole (KC); chloramphenicol (CMP); promethazine (PMZ); thioridazine (TRZ); amiodarone (AM); clomipramine (CPM) were studied and are known to induce phospholipidosis. Furthermore, seven non-steatotic compounds were selected as negative controls and include carbamazepine (CBZ), diclofenac (DFNa), indomethacin (IM), naproxen (NP), nifedipine (NIF), nimesulide (NIM) and sulindac (SUL). Further information regarding administration routes and dosage details of steatosis/phospholipidosis/non-steatosis rat *in vivo* as well as rat *in vitro* studies are available on TG-GATE database (http://toxico.nibio.go.jp).

### Animal experiments

All animal studies were conducted by the TGP group after obtaining approval from the Ethics Review Committee for Animal Experimentation of the National Institute of Health Sciences, Japan [9], [23]. The investigation conforms to the Guide for the Care and Use of Laboratory Animals (The National Academy Press, Washington, D.C., 1996). Briefly, Crj:CD (SD) male rats aged 6 weeks were given a regular chow diet. The rats were treated as described in Table S1 (low, mid and high dose groups) and sacrificed at 3, 6, 9 and 24 hours for the single dose treatment groups as well as 3, 7, 14 and 28 days for the repeated dose animals. The liver was removed surgically and samples were stored at −80°C.

### Histological patterns of fatty liver

**Figure 1:**
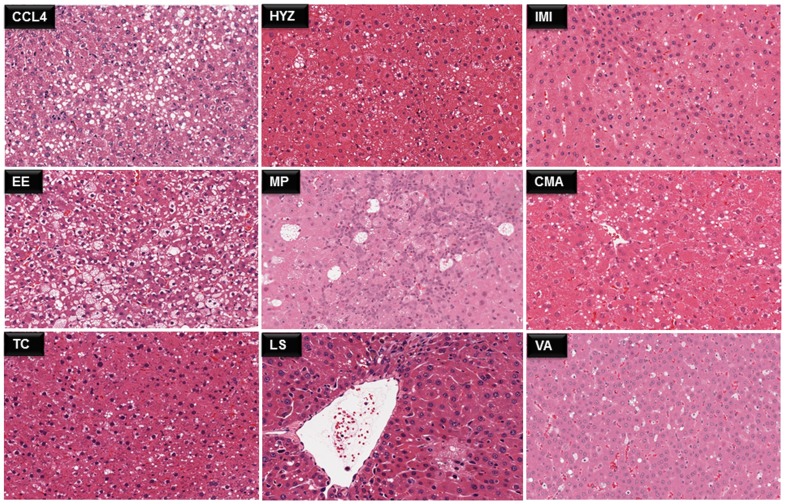
Representative images of H and E stained rat liver sections using 9 steatotic drugs. Examples of micro- or macrovesicular steatosis induced by 9 compounds after repeated high dose treatments for 28 days. Carbon tetrachloride  =  CCL4, hydroxyzine  = HYZ, imipramine  =  IMI, ethinylesterdiol  =  EE methapyrilene hydrochloride  =  MP, coumarin  =  CMA, tetracycline  =  TC, lomustine  =  LS, vitamin A =  VA. The images were retrieved from TG-GATE database (http://toxico.nibio.go.jp).

**Figure 2:**
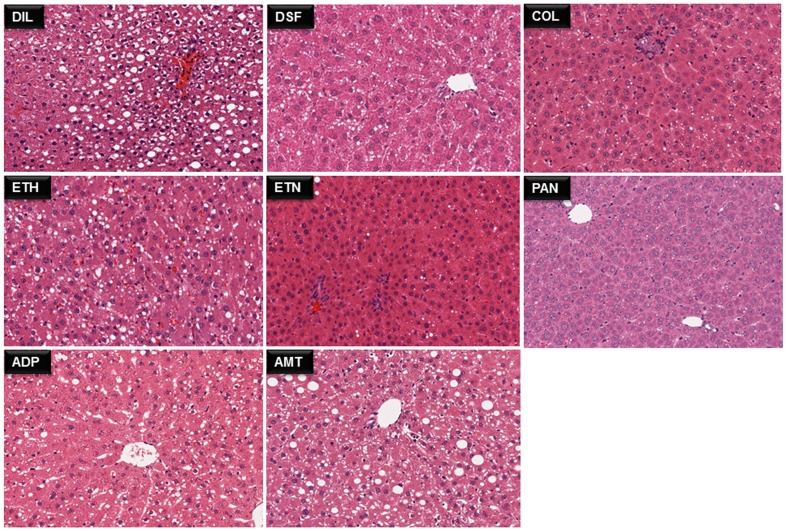
Representative images of H and E stained rat liver sections using 8 steatotic drugs. Examples of micro- or macrovesicular steatosis induced by 8 compounds after repeated high dose treatments for 28 days. Diltiazem  =  DIL, disulfiram  =  DSF, colchicine  =  COL, ethionalamide  =  ETH, ethanol  =  ETN and puromycin aminonucleoside  =  PAN, amitriptyline  = AMT. The images were retrieved from TG-GATE database (http://toxico.nibio.go.jp).

The liver sections were fixed with formalin and later dehydrated. The dehydrated samples were paraffin-embedded, sectioned to 5 µm thickness and stained with H and E. The slides were read by board certified pathologists including Dr. Kato of Shionogi pharmaceuticals after evaluating randomly selected fields at 20X magnification. Figure 1 and 2 displays liver sections of rats treated with the aforementioned 17 compounds for high dose animals treated for day 28 to evidence different grades of fatty liver disease. The images were retrieved from the TG-GATE database (http://toxico.nibio.go.jp).

### Microarray data analysis

Microarray raw data were retrieved from TG-GATE (http://toxico.nibio.go.jp/english/index.html). The data were analyzed using the robust multi-array average (RMA) methodology in the Bioconductor, R package for background-adjusted, normalized, and log-transformed perfect matched values of individual probes from the Affymetrix GeneChip Rat Genome 230 2.0 Array. Subsequently, ratios were calculated by using average signals obtained from treatment group animals divided by the average signal from control animals that received the vehicle only. The student t test was employed to calculate the adjusted *p* value with multiple testing corrections. Finally, DEGs were obtained by considering the criteria of *p* value less than 0.05 and fold change more than 1.5. Subsequent data analysis focussed on 200 genes which are mechanistically involved in hepatic steatosis and the data were compared against low, medium and high doses at different time points. Next to hypothesis driven searches for LD related gene expression changes we regarded for genes frequently altered using the following criteria: A gene needs to be significantly regulated at least 10-times either based on dose or time or its combinations amongst different drugs.

### Hierarchical gene cluster analysis

A heatmap for steatosis related DEGs was constructed based on the average-linkage hierarchical clustering with Pearson correlation distance using the software Multi Experimental Viewer (MeV) [http://www.tm4.org/mev.html].

### STRING Analysis

To determine protein-protein interactions amongst steatosis related DEGs the STRING software (v.9.1) was used.

## Results

### Effect of drugs/chemicals on gene expression profiles of genes associated with steatosis

A master list of 200 steatotic genes selected from our recent publication on the molecular pathophysiology of lipid droplet formation [17] is given in Table S2.

Whole genome microarray data was retrieved from the TG-GATE public repository. Subsequently, the DEG data were filtered for the master list of genes associated with steatosis (Table S2). To identify drug specific effects, the whole genome gene expression data with a log fold change ≥0.5 and p-value ≤0.05 were considered as the filtering criteria for any given conditions, that is drug, dose and time. Among the many significant DEGs, genes matching our master set of 200 genes were considered in detail. Gene networks were constructed and their gene frequencies were calculated. For further analysis, genes common among all dose levels and time points were considered as to identify associations linked to steatosis but are independent of drug specific effects.

### Single dose treatments

Within the single dose group animals, statistically significant genes of interest were further considered based on dose and time responses.

A dose dependent response was observed where mid and high dose group animals displayed more regulated genes. Note, for any given dose (low, mid and high) we requested a statistical significant change in expression to be included as a steatotic responsive gene. Here, a total of 41 genes were found to be significantly regulated (18 up- and 23 down-). In response to time, 26 genes were significantly regulated (10 up-, 16 down-).

### Repeated dose treatments

With respect to repeated drug treatment, 35 genes (6 up-, 29 down-) were significantly regulated at all four time points studied (3, 7, 14, 28 days). Additionally, in response to time 21 DEGs were regulated at least at three different time points (8 up-, 13 down-).

**Figure 3:**
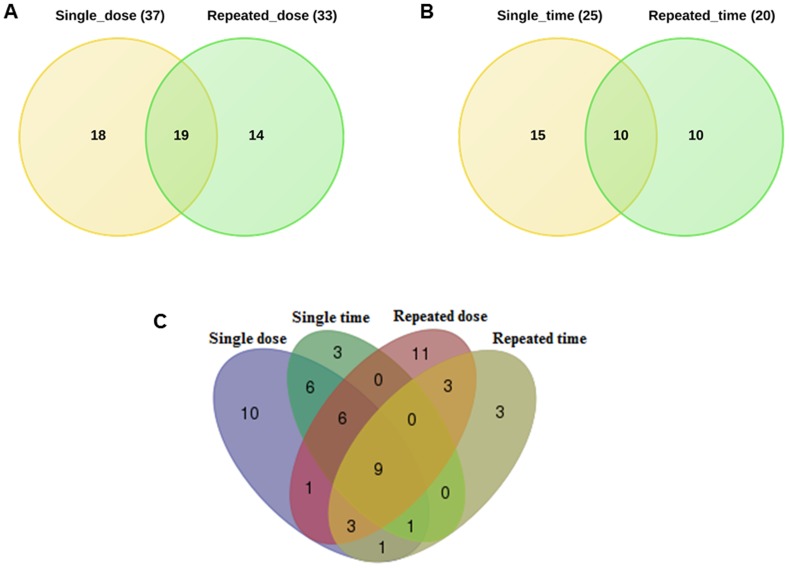
Venn diagram of DEGs based on dose and time considerations. Panel A and B represent Venn diagrams of drug induced gene expression changes after single and repeated treatment at different doses and time points, respectively. For this purpose genes regulated at different doses (low, middle and high) and/or at least at three different time points (3, 6, 9 and/or 24 h) after single treatment were considered. The same analysis was carried out for animals treated repeatedly at the low, medium and high dose for 3, 7, 14 and 28 days. Panel C combines all DEGs after single and repeated dose/time considerations. Eventually, 9 genes were found to be regulated in common.

Furthermore, to identify common DEGs we considered either dose or time constellations after single and repeated treatment of animals. The Venn diagrams depicted in Figure 3A and 3B were constructed. Our analysis revealed 19 and 10 common DEGs involved in hepatic steatosis between single vs repeated treatment conditions by considering dose and time effects, respectively (Figure 3A and 3B).

### Commonly regulated genes irrespective of time, drug and dose

The Venn diagram in Figure 3C (http://bioinformatics.psb.ugent.be/webtools/Venn/) summarises the total number of DEGs for all the conditions (single time, single dose, repeat time and repeat dose) and 9 genes were identified as commonly regulated.

In line with our expectation, genes related to glucose metabolism were regulated. Specifically angiopoietin-like 4 (ANGPTL4), glycerol kinase (GK) and pyruvate kinase (PKLR) [24]–[26] were significantly repressed and are a part of the glycolytic pathway. Conversely, induced transcript expression of the hepatic lipid transporter low density lipoprotein receptors (LDLR) was observed that leads to an increased intracellular lipid load, while, fatty acid binding protein (FABP7) was repressed indicating an alternative role in NAFLD. Fatty acid desaturases1 (FADS1) that creates a double bond in the biosynthesis of essential fatty acid was repressed as well [27] whereas the signal transducer and activator of transcription 3 (STAT3) was up-regulated and is known to play a key role in inflammation implicating its association with steatohepatitis [28]. Additionally, the serum biomarkers fibroblast growth factor 21 (FGF21) and glutamic-oxaloacetic transaminase 1 (GOT1) were significantly up-regulated in drug induced NAFLD. All the above genes are mechanistically linked to hepatic steatosis and comply with our conception and can be considered as drug induced steatosis signature genes (DIS).

**Figure 4:**
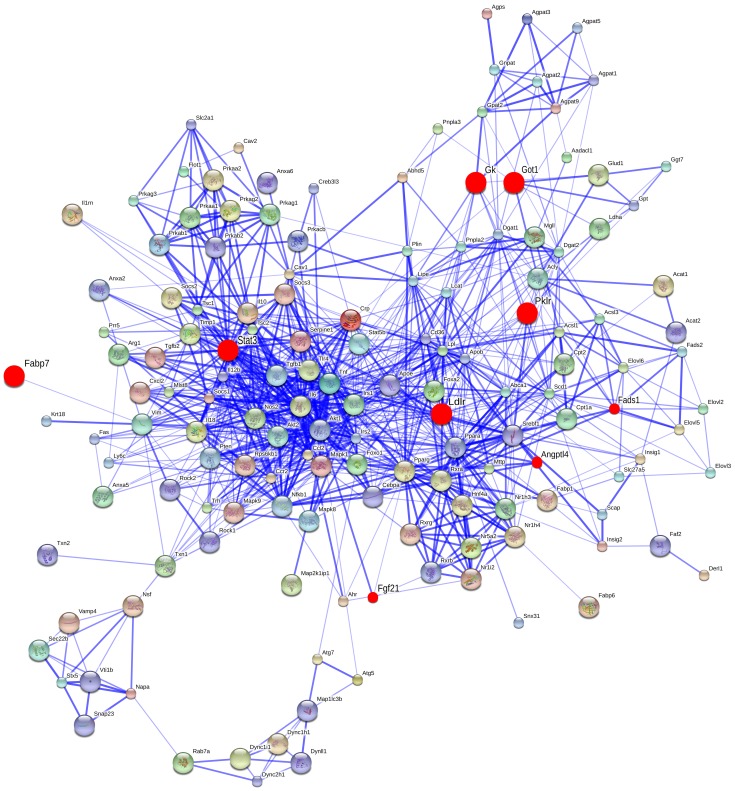
STRING network analysis. A list of 200 human steatotic genes (see Table S2) was used as input function of which 173 could be converted to rat orthologues. Eventually 150 genes interacted with each other with proven PPIs. The red circles highlight the 9 DIS signature genes commonly regulated amongst all drugs and time/dose considerations; the strength of association amongst individual partners is depicted with the thickness of the blue line (STRING version 9.1, confidence view).

To interpret functionally the significantly regulated genes, a network was constructed based on protein-protein interaction (PPI). For this purpose, the String version 9.1 (http://string-db.org/) was employed using the master list of 200 steatotic genes as input function (Table S2). Note, the 9 commonly regulated genes (marked with red colour) and their closest association partners are part of a complex PPI network (Figure 4). Among the 200 human genes associated with the pathophysiology of LD formation (see Table S2) 87% could be converted to rat orthologues to study the protein-protein interactions. As depicted in Figure 4, 150 genes are predicted for PPIs and the 9 commonly regulated genes are part of a total of 906 PPI determined for 150 genes in this network.

**Figure 5:**
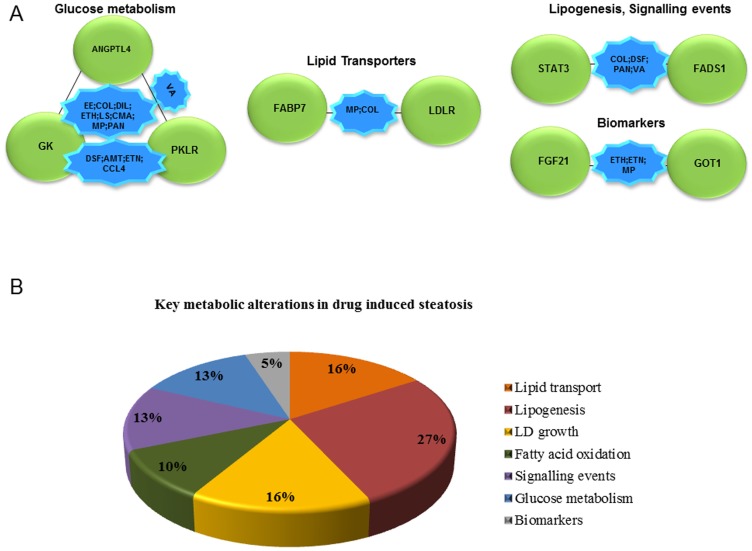
Key biological processes affected in drug induced steatosis. Panel A: The 9 DIS signature genes were categorised based on biological function by taking a total of 13 drugs/chemicals into account. Gene coding for glucose metabolism, lipid transport, and lipogeneis/signalling in addition to biomarkers are primarily affected. Panel B: The pie chart depicts the distribution of key metabolic processes based on 138 statistically significantly regulated steatotic genes in the liver of rats treated with 17 drugs.

The identified 9 common DEGs were grouped based on their biological function with respect to the 17 drugs investigated. Here, 8 compounds (EE; COL; DIL; ETH; LS; CMA; MP and PAN) were observed to cause regulation of genes involved in glucose metabolism while the compounds MP and COL displayed common regulation for lipid transporters. Likewise, 4 compounds (COL; DSF; PAN; VA) were found to modify lipogenic as well as signalling events, whereas ETH; ETN and MP were associated with induced expression of biomarkers as depicted in Figure 5A. Furthermore, among these compounds, COL and MP were identified in at least three different biological processes. The pie chart depicted in Figure 5B represents the percentage distribution of biological processes associated with drug induced steatosis and include DEGs either expressed after single or repeated treatment. Among 5 biological processes, lipogenesis was prominent (27%) followed by fatty acid oxidation (10%), lipid transport and LD growth (16%), glucose metabolism and signalling events (13%) and regulation of biomarkers (5%).

### Phospholipidosis vs steatosis

**Figure 6:**
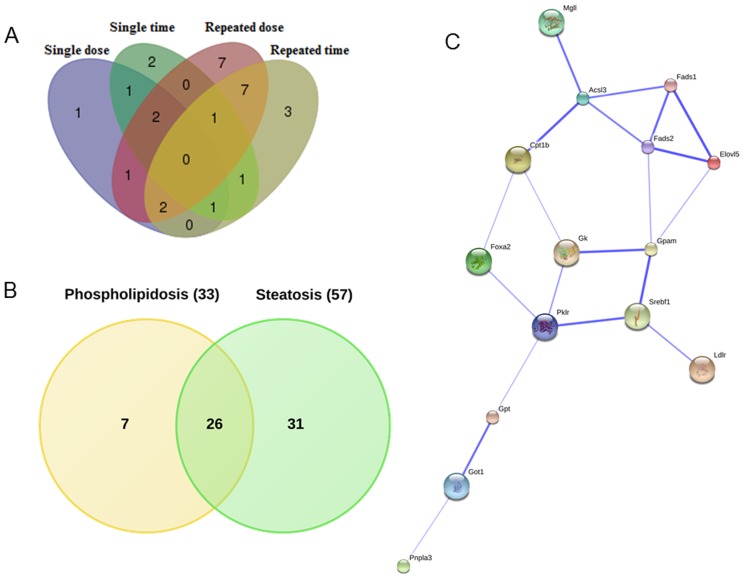
Venn diagram of DEGs associated with steatosis and phospholipidosis. Panel A: DEGs associated with phospholipidosis under all conditions (single dose at 3, 6, 9 and 24 h and repeated treatment (3, 7, 14 and 28 days) at the low, mid and high dose for 12 phospholipidosis drugs). Note, none of the DEGs were commonly regulated amongst phospholipidosis drugs. Panel B: DEGs common amongst phospholipidosis and steatosis drugs. By applying less stringent criteria (regulation in any of the conditions, either by dose or time) a total of 26 genes were commonly regulated. Panel C: PPI network among the 26 common genes. Of these 58% proteins interact with each other. The strength of association is depicted with the thickness of the blue line between the interacting proteins (STRING 9.1, confidence view).

To determine whether DEGs associated with steatosis are regulated in phospholipidosis as well, we examined gene expression data from an additional 12 drugs known for their ability to cause phospholipidosis. The treatment schedule for the phospholipidosis inducing drug is identical to that used for DIS and included HPL; PH; TMX; AM; KC; CMP; PMZ; TRZ; CPM. Histopathology revealed phospholipidosis without vacuolar formation while IMI; AMT; and HYZ were confirmed to induce both phospholipidosis as well as steatosis. To identify the subtle difference in regulation pattern between these two conditions, we analysed the transcript expression data with respect to the master list of 200 steatotic genes. Such a comparison revealed that none of the genes were commonly regulated when stringently filtered for their regulation under all 4 conditions that is dose and time from both single and repeated treatment regimens (Figure 6A). However, when gene expression changes induced by drug treatment irrespective of time and dose were considered a total of 26 genes could be defined as common amongst drugs causing steatosis and phospholipidosis (Figure 6B). The total number of DEGs from phospholipidosis data was fewer (nearly half) in comparison to DEGs from steatosis.

Furthermore, to determine possible PPIs amongst the 26 genes found to be regulated, String analysis (v 9.1) (http://string-db.org/) was employed where 15 genes were observed to interact with each other (Figure 6C).

**Figure 7:**
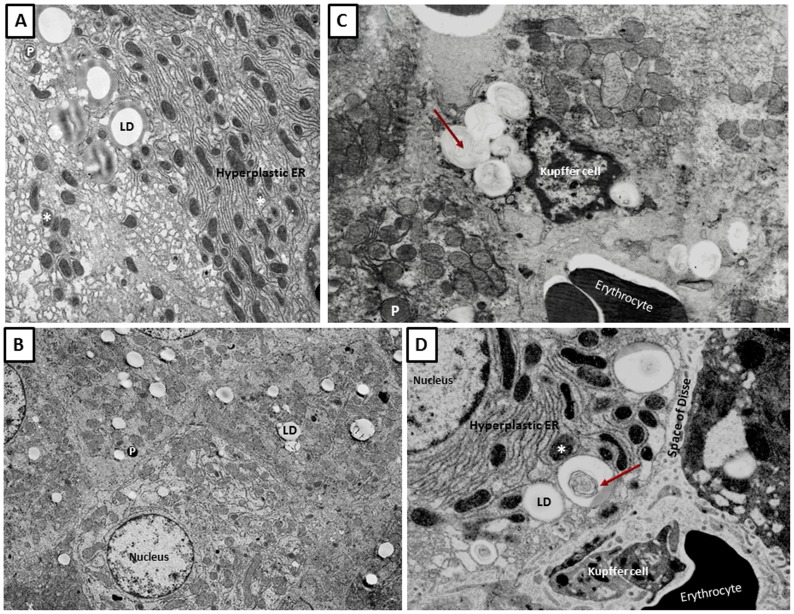
Electron microscopic images of steatosis and phospholipidosis. Depicted are examples of hepatic steatosis with lipid droplets (LD, see panel A and B). Hepatic phospholipidosis with concentric membranous structures known as lamellar bodies (arrow head) are shown in panel C and D. Note, the Kupffer cells, erythrocytes and space of Disse in panel D. LDs are also present around endoplasmic reticulum (ER, panel A and D) while mitochondria (marked with asterix) and peroxisomes (marked as P in panel B and C) appear to be in apposition ( =  close contact) with the lipid droplets and/inclusion bodies (Panel A and D). The lamellar bodies in the cytosol of Kupffer cells shown in panel C suggest their phagocytosis.

Figure 7 depicts electron microscopy images to distinguish steatosis (panel A and B) from phospholipidosis (panels C and D). Figures 7A and 7B represent steatotic condition with lipid droplets while in Figure 7C and 7D inclusion bodies with lamellar structures are shown to characterise phospholipidosis. The lipid droplets in the image appear to emerge from endoplasmic reticulum (ER) where some of LDs have close apposition with mitochondria (marked with asterix) or with peroxisomes (marked as P).

### Gene frequency distribution across different drugs, time and treatment conditions

**Figure 8:**
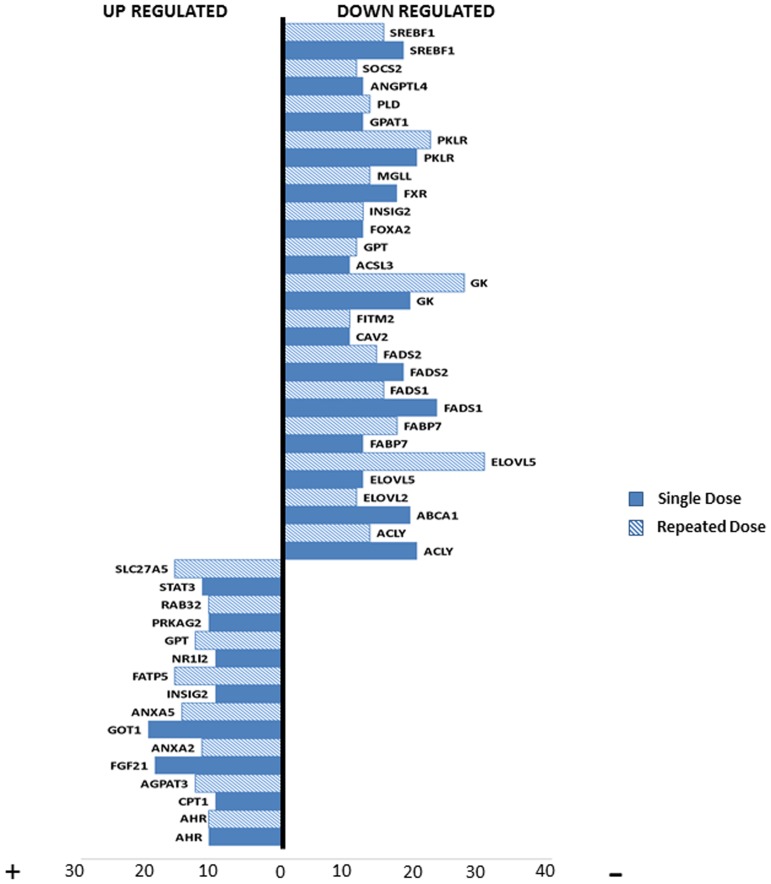
High frequency regulated genes in DIS. Individual DEGs (36 genes) with a frequency of ≥10 based on dose and time considerations are shown.

**Figure 9:**
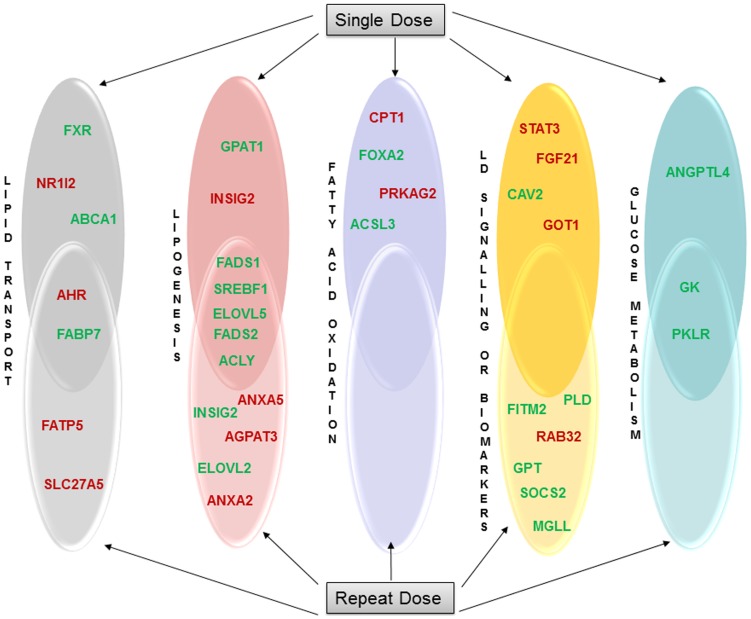
Biological processes of high frequency gene signatures. Depicted are common regulated genes (36 genes) after single and repeated treatment with high frequency using the information obtained from Figure 8. In all, 9 genes were commonly regulated with regard to their altered metabolic function.

Among the DEGs of interest, genes with a frequency of ≥10 in any dose/time constellation were compiled (Figure 8). Note, the 36 DEGs considered to be regulated at high frequency were distributed amongst a total of 12 drugs. However, this depended on the gene in question. Likewise, the 9 DEGs regulated in common (see Figure 9) were distributed amongst a total of 12 drugs. Gene appearance across the samples in Figure 8 defines aryl hydrocarbon receptors ((AHR) a lipid transport associated protein) to be commonly up-regulated in both single and repeated dose samples.

Each biological process, when considered separately, describes the significance of genes involved in causing hepatic steatosis. Figure 8 represents 14 significantly up-regulated genes that include 4 lipid transporters, 4 lipogenic genes and 1 gene linked to lipid droplet growth. Moreover, one gene coding for fatty acid oxidation and two genes involved in inflammation/signalling events were also up-regulated. Likewise, the biomarkers FGF21 and GOT1 were significantly enriched in this study.

A total of 22 genes were repressed in each of the dose groups (Figure 8). Among these, 8 genes (5-lipogenesis, 1 lipid transporter, 2 genes in glucose metabolism) showed consistently high frequency in both single and repeated dose. Additionally, in the single dose treatment animals 7 genes were repressed (2-lipid transporters, 2-fatty acid oxidation, 1 each in lipogenesis, LD growth and glucose metabolism), while for the repeated dose animals 7 genes (3-LD growth, 2-lipogenesis, 1-biomarker and 1-signalling events) were down-regulated.

Furthermore, we considered the effects of single and repeat doses among the genes with high frequency. In Figure 9 common genes with high frequency between single and repeat doses based on their biological functions are presented, 9 of which code for cellular metabolism. Among these, AHR was commonly up-regulated. The enhanced expression and nuclear translocation of AHR together with other lipid transporters augments lipid uptake in hepatocytes [29]. The lipid transporter FABP7 was repressed in both single and repeated dose group animals and in the recent study by Guzmán and colleagues LFABP was reported to be repressed during NAFLD [30]. Similar to this study we found the liver expression of FABP7 in rats [31], [32] to be significantly down-regulated during steatosis.

In our study, 5 lipogenic genes were repressed. In support, it was shown by other investigators that both FADS1 and 2 were repressed in NAFLD [33]. We observed repression of elongases V (ELOVL5) that in turn regulates sterol regulatory element binding protein 1c (SREBF1) to induce hepatic steatosis [34]. Another study suggests the transcriptional activity of SREBF1 to be enhanced in response to palmitoleate and influences the regulation of SREBF1 [35] while ATP citrate lyase (ACLY) that converts citric acid to acetyl CoA was down-regulated. The reduced expression of ACLY affects very low density lipoprotein (VLDL) secretion as well as fatty acid turnover in the liver [36]. Furthermore, GK and PKLR that play a key role in glucose metabolism were repressed in expression and their altered expression is associated with hepatic steatosis [25], [26].

Moreover, a comparison of common genes after single and repeated treatment with high frequency (≥10 fold) revealed AHR and FADS1, FABP7, GK and PKLR to be consistently regulated across the samples. In another microarray study, FADS1 was also found to be repressed where it is predicted to play a role in hepatic steatosis [33]. In agreement with reported works, our analysis also evidences significant induced expression for FADS1 and FABP7 genes, however, GK and PKLR were repressed within the same group of animals.

**Figure 10:**
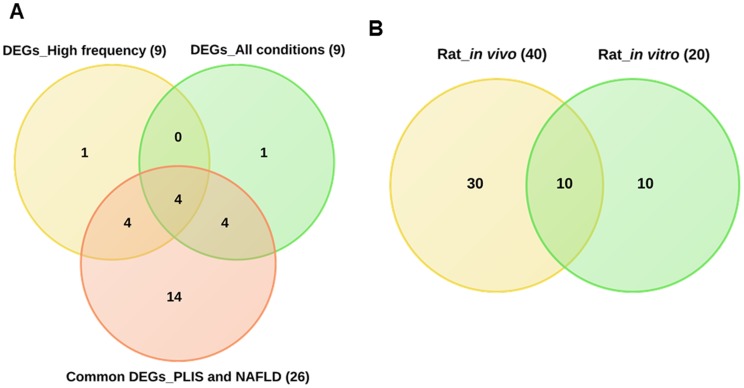
Venn diagram of genes regulated in DIS. Panel A depicts the distribution of 9 commonly regulated genes under all conditions (see also panel C of Figure 3) and were compared with frequently regulated genes depicted in Figure 9 as well as 26 DEGs common amongst drugs causing phospholipidosis and steatosis (see panel C, Figure 6). Among the three conditions, 4 genes, i.e. FABP7, FADS1, GK and PKLR were regulated alike. Panel B represents single dose (up to 24 hours) rat hepatocyte data that was compared with rat *in vivo* data under similar conditions. Here, 10 genes were in common in both of the conditions (ANGPTL4, CIDEC, CPT1, FABP7, FGF21, FOXA2, IRS2, MGLL, RXRG and VIM).

To define a gene signature predictive of hepatic steatosis, the 9 commonly regulated genes induced by steatotic drugs under all conditions (single/repeated doses and time points (refer to Figure 3C)) were compared with the genes found to be frequently regulated (see Figure 9). Between the two criteria, 4 genes, i.e. FABP7, FADS1, GK and PKLR were commonly regulated. Interestingly, these 4 genes were also present among the 26 commonly regulated genes in drug induced steatosis and phospholipidosis and therefore do not distinguish between the two conditions (Figure 10A).

### *In vitro* rat hepatocyte studies

Findings from rat hepatoctyes treated for 2, 8 and 24 hours were compared with data from rat *in vivo* studies after single treatment for 3, 6, 9 and 24 hours (Figure 10B). A total of 10 genes were regulated in common. Among these were 3 LD associated proteins, i.e. cell death inducing DNA fragmentation factor A like effector C (CIDEC), monoglyceride lipase (MGLL) and vimentin (VIM) which were repressed together with ANGPTL4 that takes part in glucose metabolism. In addition, 3 genes related to fatty acid oxidation were regulated with insulin receptor substrate 2 (IRS2) being up-regulated whereas carnitine palmitoyltransferase 1 (CPT1) and forkhead box protein A2 (FOXA2) being down-regulated, all of which are mechanistically linked to steatosis. Note, altered signalling events are associated with IRS2 post-translational modifications to subsequently down-regulate fatty acid oxidation such as CPT1 and the transcription factor FOXA2 [17]. Moreover, regulation of the cytoskeletal protein VIM is known for its significant regulation in fatty livers [37] while reduced lipolysis can be inferred based on the observed deregulation of MGLL. Lastly, genes associated with lipid transporters such as FABP7 and retinoid X receptor gamma (RXRG) were repressed, while FGF21, a biomarker candidate for NAFLD was up-regulated.

### Hierarchical gene cluster analysis

**Figure 11:**
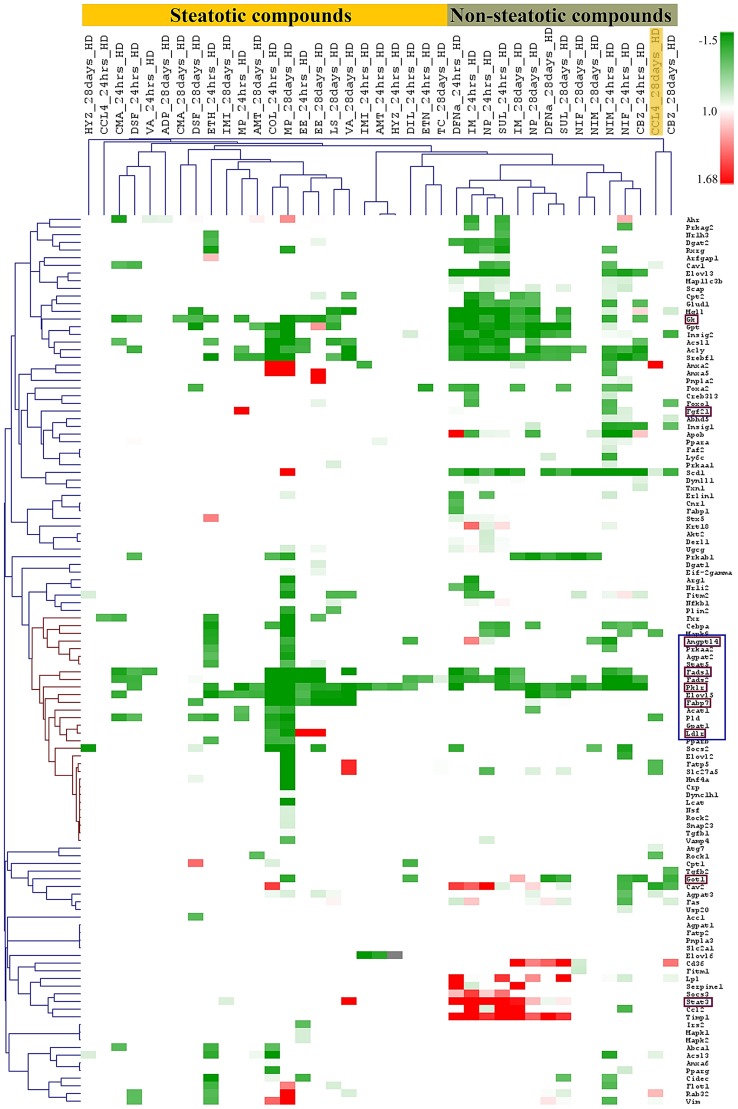
Hierarchical gene clustering. The average-linkage hierarchical clustering with Pearson correlation distance was applied. The values represent fold change of normalised DEGs of the selected 200 master genes summarised in Table S2. The data were analysed for each of the steatotic compounds at the highest dose and the 24 h time point after single dose and the highest dose after repeated treatment for 28 days. The steatosis compounds marked with a yellow bar are clearly segregated from non-steatotic compounds marked in grey. The boxes coloured in maroon represent DIS signature genes (see panel C of Figure 3) of which 5 (hallmarked with a blue box) group together.

Hierarchical gene cluster analysis was performed to identify genes regulated in common based on their similar expression using the aforementioned 17 steatotic drugs in addition to 7 compounds (negative controls) that do not cause steatosis. A heatmap for the predicted DEGs was constructed based on the average-linkage hierarchical clustering with Pearson correlation distance by using Multi Experimental Viewer (MeV) software (http://www.tm4.org/mev.html). Only high dose animals and the time points 24 hrs (single dose) and 28 days (repeated dose) for each drug/chemical was considered with a significant log fold change ≥0.5 and p-value ≤0.05 as the filtering criteria. Genes significantly regulated among the master list of 200 genes were selected. The results show a clear separation between steatosis (in yellow) and non steatosis (in grey) clustering groups, except for CCL4 at day 28 which was grouped with the non-steatotic compounds (Figure 11). The Dendogram represents the distinct gene expression patterns for the study phenotypes with respect to dose and time; note, DIS signature genes grouped together in a cluster (ANGPTL4, FADS1, PKLR, FABP7 and LDLR).

### Validation of DIS signature genes

To further validate the significance of individual DIS signature genes PubMed searches were carried out. As shown in Table 1 the entire DIS signature genes are also regulated in hepatic steatosis at the protein level as evidenced in independent studies by Western blotting and/or functional assays.

**Table 1:** Experimental evidence of DIS signature genes to be expressed at the protein level in hepatic steatosis.

Gene	Species	Type of study	Experimental evidence	Reference	Associated disease
**ANGPTL4**	mice, primary rat hepatocytes	adenovirus mediated expression/effect of chemical inducers	Western blot	[44], [45]	Hepatic steatosis
**FABP7**	mice, Hepa 1–6 cell line	miR-21 inhibitor/siRNA	Western blot	[46]	Hepatic steatosis
**FADS1**	human livers	FADS1 gene polymorphisms and its effect on human hepatic lipid composition; Lipidome analysis	Western blot	[47]	Hepatic steatosis
**FGF21**	mice/human samples/HepG2	HFD/ER stressor-tunamycin	real time qRT-PCR and Western blot	[48]	Hepatic steatosis
**GK**	rats	PUFA-depletion and its effects on glycerol metabolism	GK activity assay	[49]	Hepatic steatosis
**GOT1**	mice	HFD	real time qRT-PCR and Western blot	[50]	obesity/Hepatic steatosis
**LDLR**	mice	Dietary cholesterol exacerbates hepatic steatosis and inflammation in obese LDLR Knockout mice	histopathology, qRT-PCR, IHC and lipid analysis	[51]	Hepatic steatosis
**PKLR**	rats	HFD with Vitamin A deficiency	real time qRT-PCR	[52]	Hepatic steatosis
	mice/human liver	The lipogenic transcription factor ChREBP dissociates hepatic steatosis from insulin resistance in mice and humans	real time qRT-PCR	[53]	Hepatic steatosis
			functional assays		
**STAT3**	mice	ethanol fed/HFD diet; IL-10 deficient mice	Western blot	[54]	Hepatic steatosis

## Discussion

Steatosis is a complex condition involving a myriad of cellular responses; the disease conditions vary from simple non-progressive grade to various degrees of NASH [38]. Numerous interrelated factors are responsible for the disease progression and mechanisms involved in drug induced steatosis remain vague. Based on our recent review on lipid droplet formation in hepatocytes we identified 200 genes being mechanistically involved in the different aspects of hepatic steatosis [17]. However, their regulation in drug induced steatosis is unknown. We therefore analysed whole genome transcriptomic data deposited in the publically available Open TG-GATEs database (http://toxico.nibio.go.jp). Our study comprised 17 compounds with histopathology confirmed steatosis and of vehicle treated controls as well as 7 drugs that served as negative controls, i.e. drugs causing toxicity other than steatosis. Using such data and filtering it for DEGs linked to steatosis we aimed at deciphering the different routes adapted by drugs and chemicals and their influence in altering cellular metabolism after single and repeated treatment. Moreover, the drugs causing steatosis were compared with 12 drugs causing phospholipidosis both of which lead to lipid accumulation [22]. Based on clearly set criteria and mechanistically linked gene expression changes we also searched for a gene signature predictive of DIS that would differentiate from phospholipidosis.

Among the DEGs in DIS, 19 and 10 were commonly regulated with respect to single vs repeat treatment dose and single vs repeated time considerations, respectively. For this analysis we either considered common regulated genes at different doses (low, middle and high) or genes regulated at least at three different time points (3, 6, 9 and/or 24 h) after single treatment. The same analysis was carried out for animals treated repeatedly at the low, medium and high dose for 3, 7, 14 and 28 days. Eventually, 9 genes were commonly regulated amongst all conditions (Figure 3) while for another 9 genes regulation at high frequency (≥10 fold) across the various drug doses was noted (Figure 9). Apart from common regulations individual steatotic genes become instrumental in defining the different routes adapted by the 17 drugs/agents in causing steatosis and phenotypes of it (micro- vs macrovesicular). Additionally, by relaxing the initially set criteria we identified 26 genes regulated in common in steatosis and phospholipidosis to suggest intertwined or parallel routes adapted by hepatocytes in conditions of phospholipidosis as well as steatosis.

Notably, amongst the significantly regulated genes induced by steatotic drugs, genes of glucose metabolism, lipid transport and lipid synthesis pathway were grossly changed in expression. In line with literature findings, lipid transporters such as LDLR and AHR were up-regulated, but FABP7 was repeatedly down-regulated. Drug induced AHR apart from regulating enzymes involved in xenobiotic defence, associates with CD36 to enhance hepatic lipid influx and lowers fatty acid oxidation [17], [29]. This suggests the possibility of AHR and LDLR mediated lipid uptake in steatotic condition in addition to fatty acid diffusion across the plasma membrane. In this regard it should be noted that the AHR functions as a cytosolic transcription factor to control regulation of the so called Ah-gene battery that involves many genes coding for drug transport, detoxification and cellular repair mechanism by recognising the AHR binding sites in the promoter of targeted genes. Moreover the lipogenic genes (SREBF1 and FADS1) found to be regulated in the present study are reported to be involved in steatosis but most of these studies are related to nutritionally induced steatosis. Furthermore, regulated genes involving lipid synthesis provide valuable information on the altered molecular pathways leading to hepatic steatosis. We were particularly interested in genes that directly regulate lipid droplet growth. To our surprise, the perilipin family especially perilipin 2 and 3, which are well known to be associated with steatotic livers, were not regulated at the transcript level. Although some of the DEGs associated with LD growth, LD fusion and lipolysis were changed in expression; these genes did not show repeatability in terms of dose/time or frequency of their appearance across the samples suggesting either post translational events or involvement of alternate genes in remodelling LD growth in drug induced fatty liver.

Our analysis revealed 9 commonly regulated genes under all conditions which were further characterised by deciphering their association with other genes based on PPI networks. As depicted in Figure 4 and for the mechanistically linked steatotic genes listed in Table S2 a total of 150 are interconnected of which the DIS signature genes are part of it (marked in red). Consequently, the signature genes are of potential utility to predict drug induced hepatic steatosis in rats and were mostly repressed in transcript expression (see also Figure 5A). When compared to other compounds, colchicine and methapyrilene hydrochloride alter majorly metabolic pathways in glucose metabolism, lipid transport, lipogenesis and signalling events. Additionally, in DIS lipogenesis related genes were mostly regulated followed by DEGs of LD growth and lipid transport indicating a substantial disturbance on lipid regulation in drug induced hepatic steatosis (Figure5 B). Furthermore, a thorough literature search revealed active involvement of DIS signature genes in fatty liver disease in diverse animal/human models (see Table 1).

Importantly, when drugs causing steatosis were compared with those causing phospholipidosis (Figure 6) none of the 200 steatotic genes selected were commonly regulated. However, when DEGs were analysed at less stringent conditions (considering DEGs at any of the conditions), 26 genes were regulated in common. To understand the interaction between these genes, a network was constructed. Within the network about 50 percent interact with each other (Figure 6C) to suggest involvement of parallel routes in inducing each of these phenotypes.

Moreover, when DIS signature genes were compared with frequently regulated genes (≥10 fold, see Figure 8 and 9) FABP7, FADS1, GK and PKLR were in common. Interestingly, these genes were also regulated among the 26 common genes with drugs causing either steatosis and/or phospholipidosis to suggest their utility in predicting fatty liver (Figure 10A). Additionally, a comparison between *in vivo* and *in vitro* data (Figure 10B) informed on 10 commonly regulated genes which implies comparable pathophysiological alterations in drug induced steatosis that can be exploited in drug screening assays.

The present study infers the complexities in hepatic steatosis and among biological pathways involved glucose metabolism, lipid transport and lipogenesis was majorly affected.

Understanding the regulation of genes induced by individual drugs at varying conditions of dose and time is complex. Hence we grouped drugs based on genes linked mechanistically to steatosis. In addition, commonly regulated genes based on higher frequencies and conditions of steatosis versus phospholipidosis were also evaluated. The results suggest common routes adapted by these compounds in causing drug induced steatosis. Off target activity of drugs on mitochondrial fatty acid oxidation can be linked to different enzymes (CPT1/2, acyl-CoA synthetases) involved in mitochondrial processes leading to macrovesicular steatosis that induces severe inhibition of fatty acid oxidation [7]. Hence, categorising and comparing drugs with different grades of steatosis may be more fruitful in predicting the disease progression (Figure 11).

**Figure 12:**
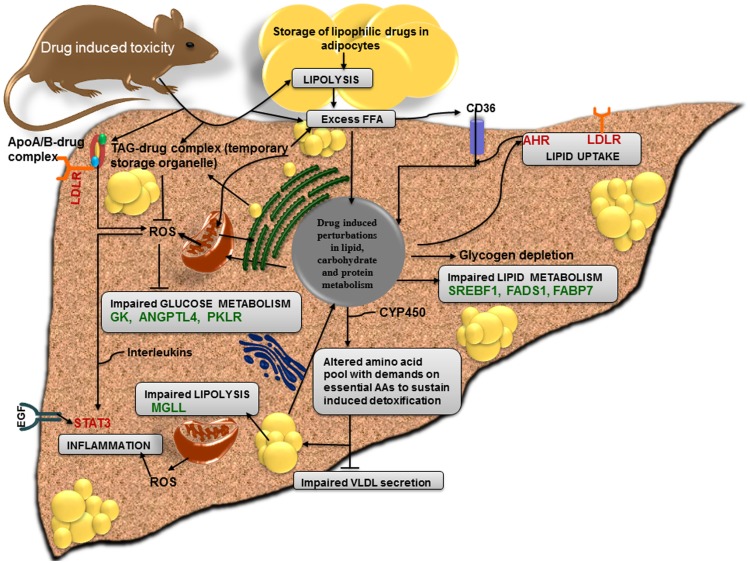
Drug induced steatosis and altered metabolic pathways. Shown is the predicted pattern of gene regulation in drug induced steatosis with respect to the 9 DIS signature genes (see panel C of Figure 3). Drug induced toxicity enhances fatty acid lipolysis in adipocytes to increase fatty acid influx. This compensates for glycogen depletion in drug induced stress conditions where fatty acids serve the energy needs in hepatocytes; however, lipophilic drugs stored in adipocytes will also become systemically available to increase the burden on drug detoxification. Besides, drug-lipoprotein-complexes enter the liver via LDLR. Insufficient drug detoxification induces ER, mitochondrial and cellular stress that leads to LD biogenesis and associated with it are altered glucose and lipid metabolism. LD may function as temporary storage organelles for drug-TAG complexes thereby reducing the burden on detoxification and ROS production. Mitochondrial dysfunction leads to ischemia and insufficient ROS detoxification propagates lipotoxicity and inflammation. To sustain detoxification amino acids (AAs) and other molecular building blocks are diverted for synthesis of induced enzymes. This in turn affects cellular homeostasis including VLDL secretion and augments lipid accumulation in drug induced steatosis.

The overall analysis showed more DEGs with respect to high dose and prolonged exposure time that substantially altered liver metabolism. Figure 12 summarises a predicted pattern of DIS induced gene regulations where drug induced toxicity stimulates lipolysis to increase fatty acid influx. As a result of drug induced stress and its associated glycogen depletion, the energy needs are met via enhanced fatty acid uptake. Additionally, the administered drug directly enters the liver via lipoprotein-lipid-drug complexes or may form triacylglycerol-drug complex to enter the lipid droplets [39]–[41]. We speculate with reason that drug induced steatosis may also be part of an adaptation process by storing lipophilic drugs in lipid droplets as temporary storage organelles thereby alleviating drug induced ER stress and its associated ROS production. Storing drugs in LDs will also prevent excessive burden on the biotransformation process to better control harmful ROS and demands on the detoxification pathway. In severely affected livers enhanced LD growth paves the way for significant changes in glucose metabolism, lipid transport and lipid metabolism. To sustain detoxification and demands on the *de novo* synthesis of detoxification enzyme the overall amino acid pool will be changed causing shortage of amino acids required for normal lipid export. A complex interplay of altered cellular signalling events may in turn decrease lipolysis (also by inhibiting lipases) and VLDL secretion in hepatocytes to further enhance lipid accumulation that perpetuates drug induced steatosis.

Evidence suggests simple low grade drug induced steatosis to be reversible and hence a benign condition but its association with other morbidities such as type 2 diabetes and/or obesity leads to higher risk for NAFLD and disease progression [42], [43]. Hence, apart from lifestyle other risk factors need to be considered to prevent drug induced steatosis.

In conclusion, our study provides insights into pathophysiological alterations in drug induced NAFLD and next to lipogenesis that was observed to be majorly altered gene regulations related to lipid transport and LD growth were affected and among the genes studied 9 were found to be regulated in common that can be explored as a signature to predict DIS.

## Supporting Information

Table S1 **List of steatotic drugs and treatment conditions.**
(DOC)

Table S2 **List of steatotic genes based on** [17] **.**
(DOC)
