# Supplementary material for: Whole Genome Transcript Profiling of Drug Induced Steatosis in Rats Reveals a Gene Signature Predictive of Outcome
Source: PLoS One. 2014 Dec 3;9(12):e114085. doi: 10.1371/journal.pone.0114085 (PMC4254931; doi:10.1371/journal.pone.0114085)
Supplement: Table S1 — List of steatotic drugs and treatment conditions. (DOC) [file pone.0114085.s001.doc]

**Supplementary Table S1: List of steatotic drugs and treatment conditions**

| ***Compound*** | ***Solvent*** | **3 -24 *hours* (in mg/kg)** | | | **3-28*days* (in mg/kg)** | | | ***Route of administration*** |
| --- | --- | --- | --- | --- | --- | --- | --- | --- |
|  |  | ***Low*** | ***Mid*** | ***High*** | ***Low*** | ***Mid*** | ***High*** |  |
| **Carbon tetrachloride (CCL4)** | Corn oil | 30 | 100 | 300 | 30 | 100 | 300 | PO |
| **Hydroxyzine (HYZ)** | 0.5%MC | 10 | 30 | 100 | 10 | 30 | 100 | PO |
| **Imipramine (IMI)** | 0.5%MC | 10 | 30 | 100 | 10 | 30 | 100 | PO |
| **Amitriptyline (AMT)** | 0.5%MC | 15 | 50 | 150 | 15 | 50 | 150 | PO |
| **Ethinylestradiol (EE)** | Corn oil | 1 | 3 | 10 | 1 | 3 | 10 | PO |
| **Methapyrilene hydrochloride (MP)** | 0.5%MC | 10 | 30 | 100 | 10 | 30 | 100 | PO |
| **Coumarin (CMA)** | 0.5%MC | 15 | 50 | 100 | 15 | 50 | 100 | PO |
| **Tetracycline (TC)** | 0.5%MC | 100 | 300 | 1000 | 100 | 300 | 1000 | PO |
| **Lomustine (LS)** | 0.5%MC | 0.6 | 2 | 6 | 0.6 | 2 | 6 | PO |
| **Vitamin A (VA)** | Corn oil | 10 | 30 | 100 | 10 | 30 | 100 | PO |
| **Diltiazem (DIL)** | 0.5%MC | 80 | 240 | 800 | 80 | 240 | 800 | PO |
| **Disulfiram (DSF)** | 0.5%MC | 60 | 200 | 600 | 60 | 200 | 600 | PO |
| **Colchicine (COL)** | 0.5%MC | 1.5 | 5 | 15 | 0.5 | 1.5 | 5 | PO |
| **Ethionamide (ETH)** | 0.5%MC | 100 | 300 | 1000 | 30 | 100 | 300 | PO |
| **Ethanol (ETN)** | dH2O | 400 | 1200 | 4000 | 400 | 1200 | 4000 | PO |
| **Puromycin aminonucleoside (PAN)** | Saline | 12 | 40 | 120 | 4 | 12 | 40 | IV |

MC: methyl cellulose; PO: pre-oral; IV: intravenous
