# Supplementary material for: Whole Genome Transcript Profiling of Drug Induced Steatosis in Rats Reveals a Gene Signature Predictive of Outcome
Source: PLoS One. 2014 Dec 3;9(12):e114085. doi: 10.1371/journal.pone.0114085 (PMC4254931; doi:10.1371/journal.pone.0114085)
Supplement: Table S2 — List of steatotic genes based on [17] . (DOC) [file pone.0114085.s002.doc]

**Supplementary Table S2: List of steatotic genes based on reference [11]**

| **Gene_Symbol** | **Name** |
| --- | --- |
| ***Lipogenesis*** |  |
| **MTORC1** | mechanistic target of rapamycin1 |
| **MTORC2** | mechanistic target of rapamycin2 |
| **PPAP2** | phosphatidic acid phosphatase |
| **ACC1** | acetyl-CoA carboxylase alpha |
| **ACAT1** | acetyl-CoA acetyltransferase 1 |
| **ACAT2** | acetyl-CoA acetyltransferase 2 |
| **ACLY** | ATP citrate lyase |
| **ANXA2** | annexin A2 |
| **ANXA5** | annexin A5 |
| **ANXA6** | annexin A6 |
| **FAS** | fatty acid synthase |
| **CNR1** | cannabinoid receptor 1 |
| **INSIG1** | insulin induced gene 1 |
| **LCAT** | lecithin-cholesterol acyltransferase |
| **FADS1** | fatty acid desaturase 1 |
| **PPARG** | peroxisome proliferator-activated receptor gamma |
| **RHEB** | Ras homolog enriched in brain |
| **SCD1** | stearoyl-CoA desaturase (delta-9-desaturase) |
| **SREBF1** | sterol regulatory element binding transcription factor 1 |
| **TSC1** | tuberous sclerosis 1 |
| **TSC2** | tuberous sclerosis 2 |
| **UGCG** | UDP-glucose ceramide glucosyltransferase |
| **DHAP-AT** | glyceronephosphate O-acyltransferase |
| **AGPS** | alkylglycerone phosphate synthase |
| **DGAT1** | diacylglycerol O-acyltransferase 1 |
| **FADS2** | fatty acid desaturase 2 |
| **AGPAT1** | 1-acylglycerol-3-phosphate O-acyltransferase 1 |
| **AGPAT2** | 1-acylglycerol-3-phosphate O-acyltransferase 2 |
| **SPT1** | serine palmitoyltransferase, long chain base subunit 1 |
| **SCAP** | SREBF chaperone |
| **MLXIPL** | MLX interacting protein-like |
| **INSIG2** | insulin induced gene 2 |
| **ELOVL2** | ELOVL fatty acid elongase 2 |
| **AGPAT5** | 1-acylglycerol-3-phosphate O-acyltransferase 5 |
| **AGPAT3** | 1-acylglycerol-3-phosphate O-acyltransferase 3 |
| **NCEH1** | neutral cholesterol ester hydrolase 1 |
| **GPAT1** | glycerol-3-phosphate acyltransferase, |
| **ELOVL5** | ELOVL fatty acid elongase 5 |
| **ELOVL6** | ELOVL fatty acid elongase 6 |
| **DGAT2** | diacylglycerol O-acyltransferase 2 |
| **CREB3L3** | cAMP responsive element binding protein 3-like 3 |
| **GPAT3** | 1-acylglycerol-3-phosphate O-acyltransferase 9 |
| **GPAT2** | glycerol-3-phosphate acyltransferase 2, |
| **S6K** | ribosomal protein S6 kinase, 70kDa, polypeptide 1 |
| **ELOVL3** | ELOVL fatty acid elongase 3 |
| ***Fatty acid oxidation*** |  |
| **CPT1** | carnitine palmitoyl transferase I |
| **CPT2** | carnitine palmitoyl transferase II |
| **ACSL1** | acyl-CoA synthetase long-chain family member 1 |
| **FOXA2** | forkhead box A2 |
| **MTP** | microsomal triglyceride transfer protein |
| **PPARA** | peroxisome proliferator-activated receptor alpha |
| **PPARB** | peroxisome proliferator-activated receptor beta |
| **FAF2** | Fas associated factor family member 2 |
| **DERL1** | derlin 1 |
| **ACSL3** | acyl-CoA synthetase long-chain family member 3 |
| ***Lipid transport*** |  |
| **PI3KC2G** | phosphatidylinositol-4-phosphate 3-kinase, type 2 alpha |
| **THR** | thyrotropin-releasing hormone |
| **ABCA1** | ATP-binding cassette, sub-family A |
| **AHR** | aryl hydrocarbon receptor |
| **AKT1** | v-akt murine thymoma viral oncogene homolog 1 |
| **AKT2** | v-akt murine thymoma viral oncogene homolog 2 |
| **CD36** | CD36 molecule |
| **FABP1** | fatty acid binding protein 1 |
| **FABP6** | fatty acid binding protein 6 |
| **FABP7** | fatty acid binding protein 7 |
| **NR5A2** | nuclear receptor subfamily 5, group A, member 2 |
| **LDLR** | low density lipoprotein receptor |
| **RXRA** | retinoid X receptor, alpha |
| **RXRB** | retinoid X receptor, beta |
| **RXRG** | retinoid X receptor, gamma |
| **NR1I2** | nuclear receptor subfamily 1, group I, member 2 |
| **FXR** | farnesoid X receptor |
| **NR1H3** | nuclear receptor subfamily 1, group H, member 3 |
| **FATP5** | Fatty acid transport protein 5 |
| **SLC27A5** | solute carrier family 27 |
| **FATP2** | Fatty acid transport protein 2 |
| ***Biomarkers*** |  |
| **GSTA** | glutathione S-transferase |
| **LAMA** | laminin, alpha |
| **LAMB** | laminin, beta |
| **LAMC** | laminin, gamma |
| **TXN1** | thioredoxin 1 |
| **COL4A4** | collagen, type IV, alpha 4 |
| **COL4A6** | collagen, type IV, alpha 6 |
| **CRP** | C-reactive protein |
| **GGT** | gamma-glutamyltransferase |
| **GLUD1** | glutamate dehydrogenase 1 |
| **GOT1** | glutamic-oxaloacetic transaminase 1 |
| **GPT** | glutamic-pyruvate transaminase |
| **KRT18** | keratin 18 |
| **LDH** | lactate dehydrogenase |
| **TIMP1** | TIMP metallopeptidase inhibitor 1 |
| **TXN2** | thioredoxin 2 |
| **FGF21** | fibroblast growth factor 21 |
| **MIR122** | microRNA 122 |
| ***LD growth*** |  |
| **DNAHC** | Dyenin |
| **KIF** | Kinesin |
| **PKA** | protein kinase, cAMP-dependent, catalytic, alpha |
| **RAB11** | Rab family protein 11 |
| **SEC22** | SEC22 vesicle trafficking protein |
| **PLIN2** | Perilipin 2 |
| **APOB** | apolipoprotein B |
| **APOE** | apolipoprotein E |
| **ARG1** | arginase 1 |
| **CAV1** | Caveolin1 |
| **CAV2** | Caveolin2 |
| **CES1** | carboxylesterase 1 |
| **DYNC1H1** | Dyenin |
| **DYNC1I1** | Dyenin |
| **PLD** | Phospholipase D |
| **LIPE** | lipase, hormone-sensitive |
| **LPL** | lipoprotein lipase |
| **NSF** | N-ethylmaleimide-sensitive factor |
| **PLIN1** | Perilipin 1 |
| **PKD** | Protein kinase D |
| **RAB5** | Rab family protein 5 |
| **SNCG** | synuclein, gamma |
| **STX5** | syntaxin 5 |
| **VIM** | vimentin |
| **RAB7** | Rab family protein 7 |
| **DYNLL1** | dynein, light chain, LC8-type 1 |
| **VAMP4** | vesicle-associated membrane protein 4 |
| **SNAP23** | synaptosomal-associated protein, 23kDa |
| **NAPA** | N-ethylmaleimide-sensitive factor attachment protein, alpha |
| **ATG5** | autophagy related 5 |
| **FLOT1** | flotillin 1 |
| **PLIN3** | Perilipin 3 |
| **VTI1B** | vesicle transport through interaction with t-SNAREs 1B |
| **ATG7** | autophagy related 7 |
| **ERLIN1** | ER lipid raft associated 1 |
| **RAB32** | Rab family protein 32 |
| **ERLIN2** | ER lipid raft associated 2 |
| **MGLL** | monoglyceride lipase |
| **RAB18** | Rab family protein 18 |
| **HILPDA** | hypoxia inducible lipid droplet-associated |
| **ABHD5** | abhydrolase domain containing 5 |
| **ARFGAP1** | ADP-ribosylation factor GTPase activating protein 1 |
| **PNPLA2** | patatin-like phospholipase domain containing 2 |
| **CIDEC** | cell death-inducing DFFA-like effector c |
| **DYNC2H1** | Dyenin |
| **PNPLA3** | patatin-like phospholipase domain containing 3 |
| **MAP1LC3B** | microtubule-associated protein 1 light chain 3 beta |
| **DNAAF1** | dynein, axonemal, assembly factor 1 |
| **FITM2** | fat storage-inducing transmembrane protein 2 |
| **FITM1** | fat storage-inducing transmembrane protein 1 |
| **PLIN5** | Perilipin 5 |
| **SEC1** | SEC1 vesicle trafficking protein |
| **PLIN4** | Perilipin 4 |
| ***Signalling events*** |  |
| **LY6C** | lymphocyte antigen 6 complex, locus C1 |
| **EIF-2ALPHA** | eukaryotic translation Initiation Factor 2alpha |
| **CXCL2** | chemokine (C-X-C motif) ligand 2 |
| **IL1** | interleukin 1 |
| **IL6** | interleukin 6 |
| **IL8** | interleukin 8 |
| **IL10** | interleukin 10 |
| **IL12** | interleukin 12 |
| **IL17** | interleukin 17 |
| **IL18** | interleukin 18 |
| **NFKB1** | nuclear factor of kappa light polypeptide gene enhancer in Bcells1 |
| **NOS2** | nitric oxide synthase 2, inducible |
| **SERPINE1** | serpin peptidase inhibitor, clade E member 1 |
| **PRKAA1** | protein kinase, AMP-activated, alpha 1 catalytic subunit |
| **PRKAA2** | protein kinase, AMP-activated, alpha 2 catalytic subunit |
| **PRKAB1** | protein kinase, AMP-activated, beta 1 non-catalytic subunit |
| **PRKAB2** | protein kinase, AMP-activated, beta 2 non-catalytic subunit |
| **PRKAG1** | protein kinase, AMP-activated, gamma 1 non-catalytic subunit |
| **MAPK2** | mitogen-activated protein kinase 2 |
| **MAPK1** | mitogen-activated protein kinase 1 |
| **MAPK8** | mitogen-activated protein kinase 8 |
| **MAPK9** | mitogen-activated protein kinase 9 |
| **PTEN** | phosphatase and tensin homolog |
| **PTGER2** | prostaglandin E receptor 2 |
| **NFKB3** | nuclear factor of kappa light polypeptide gene enhancer in Bcells3 |
| **ROCK1** | Rho-associated, coiled-coil containing protein kinase 1 |
| **CCL2** | chemokine (C-C motif) ligand 2 |
| **STAT3** | signal transducer and activator of transcription 3 |
| **STAT5** | signal transducer and activator of transcription 5 |
| **TGFB1** | transforming growth factor, beta 1 |
| **TGFB2** | transforming growth factor, beta 2 |
| **TLR4** | toll-like receptor 4 |
| **TNFA** | tumor necrosis factor |
| **SOCS1** | suppressor of cytokine signaling 1 |
| **EIF-2GAMMA** | eukaryotic translation Initiation Factor 2beta |
| **SOCS2** | suppressor of cytokine signaling 2 |
| **EIF-2BETA** | eukaryotic translation Initiation Factor 2gamma |
| **SOCS3** | suppressor of cytokine signaling 3 |
| **ROCK2** | Rho-associated, coiled-coil containing protein kinase 2 |
| **USP20** | ubiquitin specific peptidase 20 |
| **USP33** | ubiquitin specific peptidase 33 |
| **PRKAG2** | protein kinase, AMP-activated, gamma 2 non-catalytic subunit |
| **PRKAG3** | protein kinase, AMP-activated, gamma 3 non-catalytic subunit |
| **CCR2** | chemokine (C-C motif) receptor 2 |
| ***Glucose metabolism*** |  |
| **CEBPA** | CCAAT/enhancer binding protein, alpha |
| **FOXO1** | forkhead box O1 |
| **GK** | glycerol kinase |
| **HNF4A** | hepatocyte nuclear factor 4, alpha |
| **IRS1** | Insulin receptor substrate1 |
| **PKLR** | pyruvate kinase |
| **SLC2A1** | solute carrier family 2, member 1 |
| **IRS2** | Insulin receptor substrate2 |
| **ANGPTL4** | angiopoietin-like 4 |
